# Intracranial dose constraints in IDH-wildtype glioblastoma: a framework for context-specific application

**DOI:** 10.3389/fonc.2026.2003368

**Published:** 2026-10-30

**Authors:** Flavio Donnini, Paolo Tini

**Affiliations:** Unit of Radiation Oncology, Department of Medicine, Surgery and Neurosciences, University of Siena, Siena, Italy

**Keywords:** brainstem, competing risk, dose constraints, glioblastoma, hippocampus, organs at risk, radiotherapy planning, subventricular zone

## Abstract

Intracranial dose constraints are often treated as fixed properties of neural tissue, yet their meaning depends on the metric, volume definition, source population and clinical time horizon in which they were derived. In IDH-wildtype glioblastoma, these conditions may differ from current planning practice. We identify three potential discontinuities: non-equivalent dose metrics may be treated as interchangeable; volume- or subregion-specific recommendations may be collapsed into single organ-level limits; and thresholds derived in longer-surviving populations may be transferred to a disease in which competing mortality may precede several late endpoints. The brainstem illustrates the metrological problem particularly clearly, whereas the hippocampus and subventricular/periventricular regions illustrate the problem of disease-specific validation. We propose a structure-specific framework with three operations: evaluation of each constraint in its source metric and anatomical definition; classification of each objective by predefined criteria of evidentiary status, endpoint severity and latency, which separates established limits from optimisation objectives; and explicit documentation of any target-coverage trade-off. No new numerical thresholds are proposed, and prognosis never justifies exceeding an established limit. Constraints protecting against severe irreversible injury, including those for the optic nerves and chiasm, are not reassessed.

## 1 Introduction

Modern inverse planning, volumetric delivery and daily image guidance have transformed what is geometrically achievable in intracranial radiotherapy. The evidence base defining what should be avoided has evolved more slowly, and several limits in routine use remain traceable to data generated with different imaging, dose-reporting and prognostic assumptions.

This is not an argument that neural tissue tolerates more radiation than previously believed. It is an argument that a dose constraint is inseparable from the quantity measured, the volume to which it applies, the endpoint it was designed to prevent and the population in which that endpoint was observed. When any of these elements changes, the transfer should be made explicit rather than treated as biologically neutral.

The issue is particularly relevant in IDH-wildtype glioblastoma because targets often abut critical neural structures and the disease has substantial competing mortality. Some avoided toxicities occur within the expected survival of many patients; others were characterised over much longer horizons. The resulting planning problem is therefore structure- and endpoint-specific, not a general case for relaxing normal-tissue protection.

We propose a framework for newly diagnosed IDH-wildtype glioblastoma treated with conventional fractionation. Metric fidelity is implicit in existing guidance. What this Perspective adds is threefold: a provenance audit of the non-equivalent formulations that coexist for the same structures (**Table 1**), a predefined classification separating established limits from optimisation objectives, and a minimum dataset for documenting constraint-driven target compromise. No new numerical thresholds are introduced, constraints whose endpoint is severe and irreversible are not reassessed, and nothing proposed here applies to the optic nerves or chiasm.

**Table 1:** Provenance of dose constraints for intracranial structures. In this article brain denotes uninvolved brain, the organ excluding the target volume, as recommended for dosimetric assessment in this disease (11); the source publications did not always make this distinction.

Structure	Primary source	Source population and evidence	Metric as originally derived	Coexisting formulations and potential points of substitution
Brain (whole and partial), conventional fractionation	Emami 1991 (1); Lawrence 2010 (6); Lambrecht 2018 (8); Niyazi 2023 (11)	Mixed tumour types and sites, planned in two dimensions or early three dimensions without routine heterogeneity correction. Literature review supplemented by the opinion and experience of clinicians at four institutions, in the explicitly acknowledged absence of an adequate database (1). Late toxicity generally reported as a proportion of patients treated rather than of those at risk (6)	Tolerance doses assigned to nominal fractional volumes (whole, two thirds, one third), without dose-volume histograms and without a defined evaluation volume (1). European consensus proposes a volumetric limit, V60 Gy of 3 cc or less in EQD2, for symptomatic necrosis (8)	A near-maximum dose to a small volume, if used in place of the published volumetric limit, evaluates a different quantity. No threshold exists for neurocognitive endpoints, for which consensus applies ALARA (8); the glioblastoma guideline recommends subtracting the GTV from the brain contour (11)
Brain, single and multifraction stereotactic radiosurgery	Milano 2021 (7)	Brain metastases and arteriovenous malformations treated with modern radiosurgery. Fifty-one reports pooled 1995-2018; cohorts composed solely of glioma, meningioma, vestibular schwannoma or brainstem targets excluded by design	V12 of 5, 10 and more than 15 cm3, including the target volume, associated with approximately 10%, 15% and 20% risk of symptomatic radionecrosis after single-fraction radiosurgery for brain metastases	Use as a generic brain tolerance would extend it outside its source population and indication; computation after subtracting the target changes the modelled quantity, which for a 2.5 cm lesion can fall to less than half the modelled volume (7)
Brainstem	Mayo 2010 (4); Lambrecht 2018 (8); Eekers 2018 (9); Niyazi 2023 (11); Kruser 2019 (12)	Predominantly skull base, head and neck and paediatric posterior fossa series planned with three-dimensional conformal photons or particles; most enrolled fewer than one hundred patients, and patients with full neurological recovery had received more than 60 Gy to 37% of the organ on average (4). European consensus excludes reirradiation, hypofractionation and paediatric data (8)	Whole brainstem 54 Gy, with 1 to 10 mL permitted up to 59 Gy at 2 Gy per fraction or less and risk rising markedly above 64 Gy (4); D0.03 cc of 54 Gy EQD2 for the interior and 60 Gy EQD2 for the surface, contoured as distinct subregions (8, 9)	Four coexisting formulations: the two at left, plus whole-organ 54 Gy with D0.03 cc of 56 Gy (11), and a planning risk volume near-maximum below 55 Gy, with 60 and 64 Gy accepted for core and surface where gross disease involves the organ (12). A single organ-level maximum corresponds to none of them. In one glioblastoma trial, Dmax denoted the maximum dose to any volume above 1% of the organ (19)
Optic nerves and chiasm	Mayo 2010 (5); Lambrecht 2018 (8); Niyazi 2023 (11)	Skull base and sellar tumours treated with three-dimensional conformal radiotherapy. Review in which few studies contained data adequate for dose-volume modelling	Risk rises markedly above 60 Gy at approximately 1.8 Gy per fraction (5); European consensus D0.03 cc of 55 Gy EQD2 or less (8); glioblastoma guideline Dmax below 55 Gy with D0.03 cc of 56 Gy (11)	As published
Hippocampi	Gondi 2013 (13); Goda 2020 (16); operationalised in Lambrecht 2018 (8) and Niyazi 2023 (11); glioblastoma-specific associations (17–20)	Benign and low-grade tumours, in adults and in young patients respectively, treated with IMRT or fractionated stereotactic radiotherapy. Prospective cohorts of 18 and 48 patients, assessed at 18 months and at 5 years	EQD2 above 7.3 Gy to 40% of the bilateral hippocampal volume, D40% (13); mean dose above 30 Gy to the left hippocampus (16)	Adopted by consensus as its only non-near-maximum constraint, described as somewhat preliminary and glossed in the same table as the mean dose to 40% of the volume of both hippocampi (8); carried into glioblastoma with the model described as uncertain, a hard-threshold reading unsupported and coverage not to be compromised for it (11)
Subventricular zone and periventricular space	Eekers 2021 (10) for the contour; no source for a threshold	No population in which a threshold has been derived. Contoured by contemporary consensus and classified as an organ at risk on the basis of late contrast-enhancing lesions after particle therapy, while the same document identifies its innermost region as a neural stem cell niche associated with gliomagenesis (10)	Not applicable: no threshold has been proposed (10)	No consensus objective. Delineated as a 5 mm rind in the atlas (10) and as 4 mm by other groups (25); the glioblastoma guideline advises against intentional inclusion of the zone in the target volume (11)

The final column lists the formulations that coexist in current guidance and what a substitution between them would change; it does not describe how often substitutions are made. The optic apparatus is included for completeness.

Metric definitions. Dmax, point maximum dose; D0.03 cc, minimum dose to the most irradiated 0.03 cc, used as a near-maximum; Dmean, mean dose to the structure; DX%, minimum dose to the most irradiated X% of the structure volume; VX Gy, volume receiving at least X Gy. Whole-organ formulations apply to the entire contoured structure; subregion formulations apply separately to anatomically defined parts, such as brainstem surface and interior (9). PRV, planning risk volume, the organ plus a geometric margin. EQD2, equieffective dose in 2 Gy fractions, with the α/β value specified by each source. ALARA, as low as reasonably achievable.

Sources were identified by tracing each intracranial constraint in current glioblastoma guidance and European consensus to its primary publication, supplemented by the QUANTEC and HyTEC reviews. For the hippocampus and periventricular region, PubMed was also searched for dose-outcome studies in glioblastoma, retaining null or contrary findings. The search was not systematic.

## 2 The provenance of brain dose constraints

Contemporary intracranial dose constraints are an accumulation of evidence layers generated under different definitions of dose, volume and clinical risk. The age of a layer does not by itself make it inapplicable; the relevant question is whether its metric, source population and endpoint transfer to the setting in which it is applied.

The foundational compilation by Emami and colleagues assigned tolerance doses to nominal fractional organ volumes in the acknowledged absence of an adequate quantitative database, combining literature review with expert experience (1). These estimates predated routine dose-volume histograms and modern heterogeneity correction.

QUANTEC subsequently incorporated three-dimensional dose-volume and outcome data (2), while explicitly noting persistent limitations in modelling, heterogeneous reporting and interactions with systemic treatment (3). Structure-specific reviews reflected the same constraints: relatively small brainstem series (4), sparse dose-volume information for the optic apparatus (5), and cerebral endpoints whose latency is often measured in years (6).

HyTEC addressed stereotactic treatment by pooling 51 reports of symptomatic radionecrosis against irradiated volume, but cohorts composed solely of glioma, meningioma, vestibular schwannoma or brainstem targets were excluded; the model was anchored mainly in brain metastases and arteriovenous malformations (7).

These evidence layers are translated into practice by contouring and dose-constraint consensus documents (8–10) and glioblastoma-specific guidance (11, 12). The source recommendations may be volume- or subregion-specific, whereas plan evaluation may reduce them to a single organ-level value. We did not identify data on how often such substitutions occur; **Table 1** therefore describes the formulations that coexist for each structure and what a substitution between them would change, not how often it is made.

## 3 Metric incommensurability and the volume effect

A constraint is not simply a number: it is a number attached to a defined dosimetric quantity. Transfer between non-equivalent quantities requires validation of the correspondence.

The brainstem makes this problem visible. QUANTEC permits 54 Gy to the whole organ and up to 59 Gy to 1–10 mL with conventional fractionation, with risk rising markedly beyond 64 Gy (4). European guidance instead distinguishes an interior D0.03 cc of 54 Gy EQD2 from a surface value of 60 Gy EQD2 (8, 9). ESTRO-EANO reports whole-organ and near-maximum formulations (11), while NRG uses a planning-risk-volume limit and permits higher core or surface doses when gross disease involves the structure, explicitly prioritising these limits over the 95% coverage goal (12). These widely used source and consensus frameworks therefore constrain the same structure using non-equivalent quantities.

This does not imply disagreement about radiosensitivity. It shows that volume and geometry are part of the tolerance statement. The QUANTEC brainstem review itself described a volume effect and noted that historical whole-organ estimates were conservative relative to observed complication rates (4). HyTEC similarly modelled V12 including the target; subtracting the target can materially change the value being compared with that model (7).

Near-maximum D0.03 cc is itself a pragmatic surrogate, adopted partly because it is more robust than a point maximum and because older reports often lacked the information needed for formal EQD2 conversion (8). Pragmatic standardisation is valuable, but it should not be mistaken for validation that different metrics represent the same biological threshold.

The practical implication is metric fidelity: when the source recommendation is volume- or subregion-structured, the same structure should be retained during evaluation. Replacing it with a stricter or looser derivative should be identifiable in the planning record rather than hidden inside the label of dose constraint.

## 4 Competing risk in IDH-wildtype glioblastoma

Dose constraints also contain an implicit time horizon: the avoided toxicity must occur while the patient remains at risk, and its clinical burden must be weighed against what is surrendered to prevent it. In IDH-wildtype glioblastoma, that horizon cannot be assumed to match the populations from which every constraint was derived.

Radiation necrosis typically emerges on a time scale of approximately one to two years, whereas many neurocognitive endpoints have been characterised over longer follow-up; QUANTEC also noted that late toxicity was often reported as a proportion of all treated patients rather than those surviving long enough to be at risk (6). The frequently cited hippocampal dose-response for delayed recall was assessed at 18 months (13), whereas glioblastoma-specific studies have reported hippocampal dose-associated memory decline within 6 months (Section 5).

These horizons overlap only partly with glioblastoma survival. In the pivotal temozolomide trial, median overall survival was 14.6 months and two-year survival 26.5% (14). In the tumour-treating-fields trial, median survival from randomisation was 20.9 months in a selected population that had already completed chemoradiation without early progression (15). A substantial long-surviving tail therefore exists, but many patients do not reach the follow-up intervals used to define some delayed endpoints.

The distinction between endpoints matters. Radiation necrosis can occur within the survival of a substantial proportion of patients and remains clinically relevant. By contrast, the expected benefit of preventing very late graded cognitive effects may be attenuated by competing mortality at the population level. The two should not be combined under a generic concept of brain tolerance.

The transfer problem is amplified by source populations: benign and low-grade tumours for the hippocampal threshold (13), paediatric or mixed populations for parts of the whole-brain cognitive literature (6), and brain metastases or arteriovenous malformations for the stereotactic radionecrosis model (7).

Treatment context has also changed. Several widely used tolerance estimates were derived before concomitant temozolomide, tumour-treating fields and contemporary reirradiation strategies were in use. QUANTEC explicitly identified interactions between dose distribution and systemic treatment as unresolved and listed surgery, corticosteroids and antiepileptic therapy among confounders of cognitive outcomes (3, 6). The direction of any resulting bias is uncertain; the point is that the transfer is not automatically neutral.

Estimation of late toxicity is, formally, a competing-risk problem: death or progression-related mortality can remove a patient from the risk set before a delayed toxicity is observed. The planning trade-off is a separate decision problem. Competing-risk estimates can inform that decision, but they do not by themselves determine how target coverage should be balanced against toxicity. Crude toxicity proportions may underestimate risk among long survivors, whereas long-survivor incidence estimates applied without accounting for competing mortality may overstate the population-level benefit of avoidance. Competing-risk considerations therefore bear on how far evidence from longer-surviving populations transfers to glioblastoma, not on what dose an individual patient can tolerate.

Two safeguards follow. First, survival cannot be predicted reliably for an individual patient, and no individual survival estimate is used here to define a tolerance or to justify exceeding a limit. Second, the argument does not apply to early or subacute toxicities, or to late events whose severity and irreversibility justify protection regardless of prognosis.

## 5 Hippocampus and subventricular zone: a problem of validation, not of threshold

For the hippocampus and subventricular/periventricular regions, the central problem is disease-specific validation rather than evidence that an established threshold is too conservative. In adults with benign or low-grade tumours, an EQD2 above 7.3 Gy to 40% of the bilateral hippocampal volume predicted impaired delayed recall at 18 months in 18 evaluable patients (13). In a separate prospective cohort of young patients, mean dose above 30 Gy to the left hippocampus predicted decline in intelligence quotient at five years, with no corresponding right-sided association (16). The studies differ in metric, laterality, endpoint and time horizon, and neither was conducted in glioblastoma.

Glioblastoma-specific data exist but do not converge on a threshold. In a pooled prospective analysis including 14 patients with glioblastoma, a bilateral hippocampal D50% of 22.1 Gy EQD2 was associated with a 20% probability of decline in delayed recall at 6 months, although fewer than half of the glioblastoma patients completed testing at that point (17). In the main source cohort, 30 patients treated with neural progenitor cell-sparing radiotherapy, higher hippocampal and subventricular doses predicted greater verbal memory decline (18). In a secondary analysis of a randomised trial, mean left, but not right, hippocampal dose was associated with decline in Mini-Mental State Examination score among the 81 of 133 patients with complete cognitive data; patients receiving a left mean dose above 30 Gy EQD2 declined more, whereas the association with a bilateral D40% above 7.3 Gy did not reach significance (19). A cohort of 15 patients with grade 3 or 4 glioma showed no change in objective memory 3 months after radiotherapy (20). Metrics and endpoints differ, but decline may appear within the survival of most patients.

European consensus adopted the 7.3 Gy value while describing hippocampal constraints as preliminary and, within the same table, glossed D40% as a mean dose to 40% of the bilateral volume (8). ESTRO-EANO carried the value into glioblastoma but explicitly described the model as uncertain, rejected interpretation as a hard threshold and recommended contralateral sparing only when target coverage is preserved (11). This is precisely the distinction between recording and optimising a dose objective and treating it as a validated disease-specific tolerance.

Two claims should therefore be kept separate. Hippocampal avoidance is a justified optimisation goal, and hippocampal dose can often be reduced substantially without compromising target coverage, although less so for large targets and sometimes at the cost of higher doses elsewhere (19, 21). A validated numerical dose-response relationship in glioblastoma does not yet exist, and the consensus D40% threshold was not reproduced when tested in this disease (19). In practice, the hippocampi should be contoured, contralateral dose optimised once target coverage and established limits are met, and the achieved dose recorded in the source metric, with a value such as D40% of 7.3 Gy EQD2 treated as a reporting benchmark rather than a pass/fail criterion. Given the early decline reported, this objective is not classified as long-latency (Section 6).

The subventricular zone presents a different uncertainty. It is both a neurogenic niche and a proposed reservoir of glioblastoma-propagating cells, and published associations between incidental dose and outcome are inconsistent (22, 23); a meta-analysis found higher ipsilateral dose associated with longer progression-free but not overall survival (24), while higher dose has also been associated with verbal memory decline (18).

Contouring is also non-uniform. The EPTN atlas defines a 5 mm periventricular space, identifies its innermost component as the subventricular zone and proposes no numerical dose constraint (10). Other work has used a 4 mm rind (25), while alternative definitions of subventricular-zone involvement differ in reproducibility and prognostic association (26). The glioblastoma guideline advises against intentional inclusion of the zone in the target (11).

The periventricular region is thus a boundary case: it is contoured as an organ at risk (10), but there is no agreed direction for the desirable dose. Only fidelity to its anatomical definition and documentation apply; classification yields no threshold (**Table 2**).

**Table 2:** Structure-specific application framework.

Structure	Category and evidence	Planning status	Role of prognosis, if any, when coverage conflicts	Minimum documentation
Brain, corpus callosum, temporal lobes	Radiation necrosis: established volumetric limit; discrete, potentially severe endpoint, not long-latency. Diffuse cognitive effects: optimisation objective (ALARA); graded, long-latency endpoint; no validated GBM-specific numerical threshold.	Minimise dose and apply established volumetric limits in their source metric; do not convert ALARA objectives into unstated hard thresholds.	Necrosis limits: none. Diffuse cognitive objectives: may inform priority, with documentation; never alters a limit or defines a tolerance.	Constraint/objective, metric, evaluation volume, and quantified change in target coverage or dose if compromise occurs.
Brainstem	Established limit; severe irreversible endpoint. Tolerance evidence is volume- and subregion-dependent across source frameworks.	Evaluate using the published whole-organ, absolute-volume or subregion formulation rather than a single undifferentiated organ maximum.	None. Severe endpoint and current consensus priority are retained.	Exact metric and anatomical definition used; any local derivative from the source formulation; target compromise if present.
Hippocampi	Optimisation objective; graded endpoint, not long-latency in GBM, with decline reported within 6 months. Source thresholds are disease-external; GBM-specific associations use non-convergent metrics and endpoints.	Contour; optimise contralateral dose after target coverage and established limits; record achieved dose in the source metric; treat numerical values as reporting benchmarks, not automatic reasons to reduce target coverage.	None.	Contour definition, metric, source of any numerical objective, achieved dose, and any target-coverage trade-off.
Subventricular zone and periventricular space	No threshold. Boundary case: contoured as an organ at risk, but the direction of the desirable dose is uncertain (neurogenic niche and proposed tumour reservoir).	Apply the atlas definition used; spare when achievable without cost to target coverage.	Not applicable: no objective to weight.	Contour definition and rind width, achieved dose, and any target-coverage trade-off.
Optic nerves and chiasm	Established limit; catastrophic and irreversible visual endpoint.	No reassessment; retain established constraints.	None.	Standard constraint documentation; record any target compromise required to preserve the optic pathway.

Categories follow the criteria defined in Section 6. Prognosis does not define tolerance or prescribe plan changes; it may inform, with documentation, the priority of optimisation objectives with graded, long-latency endpoints that genuinely conflict with target coverage, and no row authorises exceeding an established limit. Constraints protecting against severe irreversible injury retain priority independently of prognosis.

Sparing these regions remains reasonable when achieved without cost. What is not justified is allowing an unvalidated numerical threshold, or a non-equivalent anatomical definition, to automatically supersede target coverage.

## 6 A context-specific framework

Clinical context already influences radiotherapy intensity in glioblastoma: abbreviated schedules are established for selected older patients (27, 28). The question here is narrower: how to make OAR-versus-target trade-offs transparent without converting prognosis into a new tolerance threshold.

Existing guidance already contains a hierarchy. ESTRO-EANO identifies structures whose limits may justify compromise of target coverage and others for which such compromise is discouraged; NRG similarly prioritises brainstem and optic constraints over the 95% coverage goal (11, 12). European consensus further states that constraints are not absolute values and should be interpreted alongside age, comorbidity and life expectancy (8). The missing elements are explicit classification of why a constraint carries that weight in glioblastoma and documentation of what is surrendered when it prevails.

We therefore propose three operations. First, preserve metric fidelity: evaluate each constraint in the dose-volume or anatomical formulation used by its source whenever feasible. A stricter local derivative may still be chosen, but it should be recognised as such.

Second, classify each objective using predefined criteria. Evidentiary status: an established limit is one that current consensus presents as a dose constraint with a specified metric and anatomical definition, derived from dose-volume-outcome data; an optimisation objective is one that the source itself describes as preliminary, uncertain, as low as reasonably achievable (ALARA) or subordinate to target coverage, or whose dose-response has not been independently reproduced; a structure without any proposed value has no threshold. Severity: severe irreversible injury is a discrete event causing permanent loss of a major neurological function, such as visual loss; a graded endpoint is a continuous or ordinal functional measure, such as neurocognitive test performance. Latency: an endpoint is long-latency when its typical onset, or the assessment horizon used to derive its threshold, exceeds the median overall survival of the population concerned. Anchoring these categories to the sources’ own labels limits subjective judgement.

Prognosis may enter the weighting of an objective only when all three conditions hold: optimisation objective, graded endpoint and long latency. It may then inform, with documentation, the priority of that objective when it genuinely conflicts with target coverage, as European consensus already allows (8); otherwise it has no role. No element of this framework authorises exceeding a limit that current consensus presents as a constraint; where such a limit conflicts with coverage, existing consensus priorities apply unchanged. Prognostic factors such as age, performance status, extent of resection and MGMT promoter methylation describe population-level risk and are not normal-tissue tolerance biomarkers (29, 30).

Third, document the trade-off. If target coverage is reduced to meet a constraint or planning objective, the record should specify the structure, metric, source or institutional rule, and the change in target coverage or dose that resulted. Without this information, the clinical consequences of constraint-driven compromise cannot be audited or prospectively studied (31).

**Table 2** applies these criteria to each structure. Its purpose is to separate established protection from optimisation and to make the cost of any compromise measurable.

## 7 Discussion

The strongest counterargument is that conservative neural constraints are appropriate because severe injury may be irreversible and uncertainty should favour safety. Some glioblastoma data support current practice: in a randomised-trial analysis, doses within protocol limits were associated with the absence of grade 2 or higher toxicity across organs at risk (19). We agree where the endpoint is catastrophic or the tolerance evidence is established. The concern is different: when evidence is weak, disease-external or metrically non-equivalent, an undocumented sacrifice of target coverage is not synonymous with evidence-based conservatism.

The framework has important limitations. Prognosis is probabilistic, not individual; no baseline model predicts survival with sufficient certainty to justify patient-specific tolerance limits. The literature discussed here was not identified systematically, the classification criteria have not been tested for inter-observer agreement, and the framework itself is conceptual rather than validated. It therefore cannot establish that changing the priority of any existing objective improves outcome.

It does, however, generate testable next steps. Prospective planning studies could record every constraint-driven target compromise using a standard dataset. Late toxicities could then be analysed with time-to-event and competing-risk methods that define the population still at risk at each horizon. Finally, hippocampal and periventricular dose-response relationships require validation specifically in molecularly defined glioblastoma, with consistent contours, metrics, neurocognitive endpoints and quality-of-life assessment.

Three principles follow. First, evaluate each constraint in the metric and anatomical definition used by its source. Second, distinguish established toxicity limits from optimisation objectives using explicit criteria. Third, document every constraint-driven compromise of target coverage. For the optic pathway and other structures at risk of severe irreversible injury, the framework changes nothing.

## Data Availability

The original contributions presented in the study are included in the article/supplementary material. Further inquiries can be directed to the corresponding author.
